# Clinical effectiveness of theobromine, Remin Pro, and their combination in managing dentin hypersensitivity: a randomized clinical trial

**DOI:** 10.1186/s12903-026-09869-0

**Published:** 2026-10-02

**Authors:** Ebtsam E. Kassab, Maha A. Niazy, Mohamed A. El Yasky, Shimaa M. Ameen

**Affiliations:** 1 Faculty of Dentistry, Fayoum University, Fayoum, Egypt; 2 Faculty of Dental Medicine for Girls, Al-Azhar University, Cairo, Egypt

**Keywords:** Dentin hypersensitivity, Theobromine, Hydroxyapatite, Remineralizing agents, Pain assessment, Randomized clinical trial

## Abstract

**Background:**

Dentin hypersensitivity is a prevalent and debilitating condition affecting 10–30% of the adult population worldwide, yet the comparative short-term clinical effectiveness of emerging natural and remineralizing desensitizing agents remains incompletely characterized. This study evaluated and compared the clinical effectiveness of theobromine gel, a hydroxyapatite-fluoride-xylitol remineralizing paste (Remin Pro), and their combination in the management of dentin hypersensitivity.

**Methods:**

A single-center, double-blind, randomized clinical study was conducted in 171 teeth from 20 patients with confirmed dentin hypersensitivity, randomly allocated to three equal groups (*n* = 57 per group) receiving either theobromine gel, a hydroxyapatite-fluoride-xylitol remineralizing paste (Remin Pro), or their combination. Three applications were administered sequentially at baseline, one week, and one month. Pain intensity in response to tactile, evaporative, and cold-thermal stimuli was assessed at four time points: before treatment, immediately after the first application, after the second application at one week, and after the third application at one month, using the Verbal Rating Scale and Visual Analogue Scale. Data were analyzed using nonparametric tests and are reported as median and interquartile range.

**Results:**

All three treatment modalities produced a statistically significant, progressive reduction in dentin hypersensitivity over time. Theobromine demonstrated significantly greater pain reduction than Remin Pro after the first and second applications, most consistently in response to evaporative and cold-thermal stimuli. After the third application, all three groups achieved broadly comparable pain reduction, with no statistically significant differences in tactile stimulation or overall pain scores.

**Conclusions:**

Within the limitations of this short-term study, Theobromine gel provides clinically faster desensitization than a hydroxyapatite-fluoride-xylitol remineralizing paste after initial applications, a distinction of practical relevance for patients requiring prompt pain relief. After three sequential applications, all agents achieve comparable clinical effectiveness, confirming that multi-application protocols are the standard of care regardless of agent selection. These findings support the use of theobromine as a viable, biocompatible alternative for the short-term management of dentin hypersensitivity and justify further investigation in long-term, placebo-controlled randomized trials.

**Trial registration:**

ClinicalTrials.gov, NCT04340869. Registered March 2, 2020.

**Supplementary Information:**

The online version contains supplementary material available at https://doi.org/10.1186/s12903-026-09869-0.

## Introduction

Dentin hypersensitivity is a short, sharp pain originating from exposed dentin in response to non-noxious thermal, evaporative, tactile, osmotic, or chemical stimuli, not attributable to any other dental pathology or defect [1]. It is among the most common complaints in dental practice, affecting approximately 10–30% of the adult population worldwide, and can significantly impair quality of life by interfering with eating, drinking, speaking, and oral hygiene [2].

The pathophysiology of dentin hypersensitivity is most comprehensively explained by Brännström’s hydrodynamic theory: external stimuli induce rapid fluid displacement within exposed dentinal tubules, activating A-delta mechanoreceptors in the pulp-dentine complex and producing the characteristic sharp, transient pain [3]. Accordingly, desensitizing therapies are broadly classified by their primary mechanism of action into two categories: (i) neural depolarizers such as potassium salts that reduce A-delta nerve fiber excitability by elevating extracellular potassium concentrations at the pulpal nerve membrane, and (ii) tubule-occluding agents including oxalates, calcium fluoride (CaF₂), hydroxyapatite, and theobromine that physically reduce hydraulic conductance by sealing open dentinal tubule orifices and thereby blunting the hydrodynamic stimulus [4, 5].

Several tubule-occluding modalities have been clinically evaluated. Calcium fluoride (CaF₂), formed at the dentin surface through the reaction of topically applied fluoride with calcium ions in dentinal fluid, acts as a sustained fluoride reservoir and reduces dentinal fluid flow via mineral precipitate formation in the tubule orifices [6]. Nano-hydroxyapatite-based formulations have similarly demonstrated clinical efficacy in reducing dentin hypersensitivity by forming a biomimetic mineral layer over exposed dentinal tubules [7]. Hydroxyapatite-fluoride-xylitol paste (Remin Pro; VOCO GmbH; Cuxhaven, Germany) hereafter referred to as Remin Pro exploits the combined remineralizing action of these three components: hydroxyapatite promotes surface remineralization through chemical affinity with the native mineral phase of dentin; fluoride reinforces tubule sealing by promoting fluorapatite and calcium-phosphate precipitate deposition [6]; and xylitol supports remineralization by stimulating salivary flow and reducing the acidogenic potential of oral biofilms [8].

Theobromine, a naturally occurring methylxanthine alkaloid in cacao (*Theobroma cacao*), has attracted increasing interest as a biocompatible desensitizing agent. Unlike caffeine and theophylline, theobromine exhibits negligible toxicity at therapeutic concentrations and has been reported to promote the formation of calcium-phosphate crystallites larger than conventional hydroxyapatite, potentially enhancing dentin hardness, resistance to acid demineralization, and the efficiency of dentinal tubule occlusion [9]. In vitro scanning electron microscopic studies have confirmed effective tubule occlusion following application of theobromine-containing formulations, with superior maintenance of the mineral plug under acidic challenge compared with fluoride alone [10].

Despite this growing body of laboratory evidence, a clear gap exists in the clinical literature. No published randomized clinical trial has directly compared theobromine with a contemporary hydroxyapatite-based remineralizing paste under a standardized multi-stimulus, multi-application protocol, nor has the potential additive or synergistic effect of combining these two mechanistically complementary agents been clinically investigated. The present study was therefore designed to address this gap directly.

This study aimed to evaluate and compare the clinical effectiveness of theobromine gel, Remin Pro, and their combination in managing dentin hypersensitivity over a 1-month observation period, using standardized tactile, evaporative, and cold-thermal stimuli and dual validated pain scales. Based on the foregoing mechanistic rationale, the null hypothesis (H₀) was that there would be no statistically significant difference in the degree of dentin hypersensitivity reduction among the three tested desensitizing agents at any assessment time point immediately after the first application, after one week, or after one month.

## Materials and methods

### Study design and reporting

This single-center, double-blind, randomized clinical study was designed, conducted, and reported in accordance with the Consolidated Standards of Reporting Trials (CONSORT) 2010 guidelines. The study assessed and compared the clinical effectiveness of three desensitizing agents for the management of dentin hypersensitivity over a one-month follow-up period.

### Ethical approval and trial registration

The study protocol, patient information sheet, and informed consent form were reviewed and approved by the Ethical Research Committee of the Faculty of Dental Medicine for Girls, Al-Azhar University, Cairo, Egypt (approval code: REC-OP-26-01; date of approval: 26 January 2020). The study complied with the principles of the Declaration of Helsinki (2013 revision). Written informed consent was obtained from all participants prior to enrollment. The trial was prospectively registered at ClinicalTrials.gov (identifier: NCT04340869; registered on 2 March 2020, prior to enrollment of the first participant; study start date: 1 August 2020, corresponding to protocol development and site preparation).

### Sample size calculation

Sample size was calculated using G*Power (version 3.1.9.7; University of Kiel; Kiel, Germany) [11], with a two-sided α of 0.05, 80% power, and an effect size of 0.682 derived from a comparable prior study [12]. The minimum required sample size was 24 teeth (8 per group). To account for potential dropouts, 171 hypersensitive teeth (57 per group) from 20 patients were enrolled.

### Materials

The materials utilized in this study, including their composition, manufacturers, and batch numbers, are summarized in Table 1.

**Table 1:** Materials used in the study

Material	Composition	Manufacturer	Batch no.
Theobromine (500 mg/L gel) (pH 7)	1000 mg of theobromine Powder, 200 ml of deionized water, 20 g of Carboxy-methyl cellulose.	Nano Gate Company, Egypt.	www.nanogate-eg.com
Remin Pro (A water-based cream)	Hydroxyapatite (calcium and phosphate), fluoride (1450 ppm), and xylitol.	VOCO GmbH, Cuxhaven, Germany.	VC 70 002002 E1 1117 V
Fine Etch (Gel form)	37% Phosphoric acid gel	SPIDENT CO., LTD., Korea	FE18222

### Preparation of materials

To prepare 200 mL of theobromine gel at 500 mg/L, 1000 mg of theobromine powder (Nano Gate Company; Cairo, Egypt) was weighed on an analytical balance and dissolved in 200 mL of deionized water using a mechanical homogenizer (20,000 rpm for 30 min). Carboxymethyl cellulose (CMC; 20 g; Sigma-Aldrich; St. Louis, MO, USA) was added gradually under continuous homogenization to obtain a lump-free gel. The pH was adjusted to 7.0 at 37 °C using a calibrated digital pH meter (accuracy ± 0.01; Mettler Toledo; Columbus, OH, USA) and monitored at weekly intervals throughout the study; the pH remained stable at 7.0 ± 0.1 across all time points. Gel preparations were stored at room temperature (25 °C) in sealed, light-protected containers. For the combination group, equal volumes of freshly prepared theobromine gel and Remin Pro were mixed immediately before each application using sterile disposable instruments.

### Eligibility criteria

Participants were selected from the outpatient clinic of the Faculty of Dental Medicine for Girls, Al-Azhar University. Inclusion criteria were: adults aged 18–35 years, in good systemic health, presenting with at least one tooth exhibiting dentin hypersensitivity associated with exposed buccal cervical dentin, and providing written informed consent. Exclusion criteria were: chronic systemic conditions affecting pain perception; teeth with active carious lesions, deep periodontal pockets (> 4 mm), or structural defects; presence of partial dentures, orthodontic appliances, dental implants, or poorly maintained restorations; known allergy to any study material; receipt of desensitizing treatment or tooth bleaching within 8 weeks before screening; and regular use of analgesic or anti-inflammatory medications [13, 14].

### Participant preparation

Before the intervention, all participants received a standardized plaque-control program including oral hygiene instruction, professional scaling, and root planning. They were instructed to use a soft-bristled toothbrush twice daily and to refrain from using desensitizing or whitening products and dietary acids during the study period. All participants were trained before baseline assessment in the use of both pain assessment instruments [15, 16].

### Grouping of teeth and assessment timeline

A total of 171 hypersensitive teeth were included and allocated to three groups (*n* = 57 per group) according to the desensitizing agent used: theobromine gel (M1), Remin Pro (M2), or a combination of theobromine and Remin Pro (M3). Hypersensitivity was assessed at four time points: baseline before treatment (T0), immediately after the first application (T1), after 1 week following the second application (T2), and after 1 month following the third application (T3).

### Randomization and allocation concealment

A computer-generated sequence was used to perform randomization in Microsoft Excel. Allocation sequences were generated by an independent investigator who did not participate in patient recruitment or outcome assessment. Allocation concealment was achieved using sealed, opaque envelopes that were opened only at the time of participant assignment.

### Blinding

This study was a double-blind clinical trial. Participants were unaware of their group assignment throughout the study. The outcome assessor, a single experienced clinician calibrated prior to the study, was blinded to treatment allocation and did not participate in treatment delivery or randomization. Calibration was performed on patients not enrolled in the study; intra-examiner reliability was quantified using Cohen’s weighted kappa (κ = 0.85), indicating strong agreement. The primary investigator, who prepared and administered the treatments, could not be blinded because of the visible differences between agents but played no role in outcome assessment.

### Treatment procedures

The treatment area was isolated using cotton rolls, cheek retractors, and high-volume suction. All treatment procedures were performed exclusively by the primary investigator. The assigned desensitizing agent was applied to the exposed dentin surface with a disposable plastic syringe and left in situ for 15 min; participants were then instructed to distribute the residual material intraorally for an additional 15 min. Participants were then advised to refrain from rinsing, eating, or drinking for 30 min. This protocol was applied identically at three visits: at baseline, before treatment (first application; T0), after 1 week (second application; T2), and after 1 month (third application; T3) [17].

### Assessment of dentin hypersensitivity

Dentin hypersensitivity was assessed at all four time points by the blinded outcome assessor using the same teeth identified at baseline. The Verbal Rating Scale (VRS) quantified pain intensity on an ordinal scale of 0 (no discomfort) to 4 (severe pain persisting after stimulus removal). Three standardized stimuli were applied sequentially, from least to most intense, with a minimum inter-stimulus interval of 2 min to prevent sensory summation [18, 19]:

(i) Tactile stimulus: a dental explorer lightly drawn across the exposed dentin surface;
(ii) Evaporative (air blast) stimulus: 1-second application of compressed air at approximately 1 cm from the dentin surface, perpendicular to the long axis, with adjacent teeth shielded;
(iii) Cold-thermal stimulus: freshly prepared ice applied to the exposed dentin surface for 3 s [20, 21].

The Visual Analogue Scale (VAS), a 100-mm horizontal line anchored by ‘no pain’ (0 mm) and ‘worst imaginable pain’ (100 mm), was used concurrently to capture pain intensity. Participants were trained in VAS use prior to baseline assessment [22].

### Statistical analysis

Statistical analysis was performed using R software (version 4.3.2; R Foundation for Statistical Computing; Vienna, Austria). All outcome variables (VRS and VAS scores) were treated as ordinal data. Normality was assessed using the Shapiro-Wilk test and was found to be violated for all outcomes and groups (*p* < 0.05). Descriptive statistics are reported as median [interquartile range (IQR): Q1–Q3]. For intergroup comparisons at each time point, the Kruskal-Wallis H-test was used, followed by Dunn’s post-hoc test with Bonferroni correction. For intragroup comparisons over time, Friedman’s test was applied, followed by pairwise Wilcoxon signed-rank tests with Bonferroni correction. The significance level was set at α = 0.05 for all analyses. Categorical variables were expressed as frequencies and percentages and analyzed using the chi-square test.

## Results

### Participant and tooth characteristics

A total of 171 hypersensitive teeth from 20 patients were included in the final analysis, with 57 teeth per treatment group. All participants were female. Anterior teeth predominated across all groups. No statistically significant differences were observed among groups for tooth type or arch distribution (*p* > 0.05), confirming baseline comparability of tooth characteristics. It should be noted, however, that baseline VRS and VAS pain scores differed significantly among the three groups (*p* < 0.001 for all outcomes; Tables 2, 3, 4 and 5). This baseline inequality is an artifact of the limited patient numbers and clustering of multiple teeth within patients and does not reflect selection bias, as allocation concealment was maintained throughout; however, it is an important caveat for interpreting intergroup comparisons at subsequent time points.

### Clinical assessment of dentin hypersensitivity

**Fig. 1:**
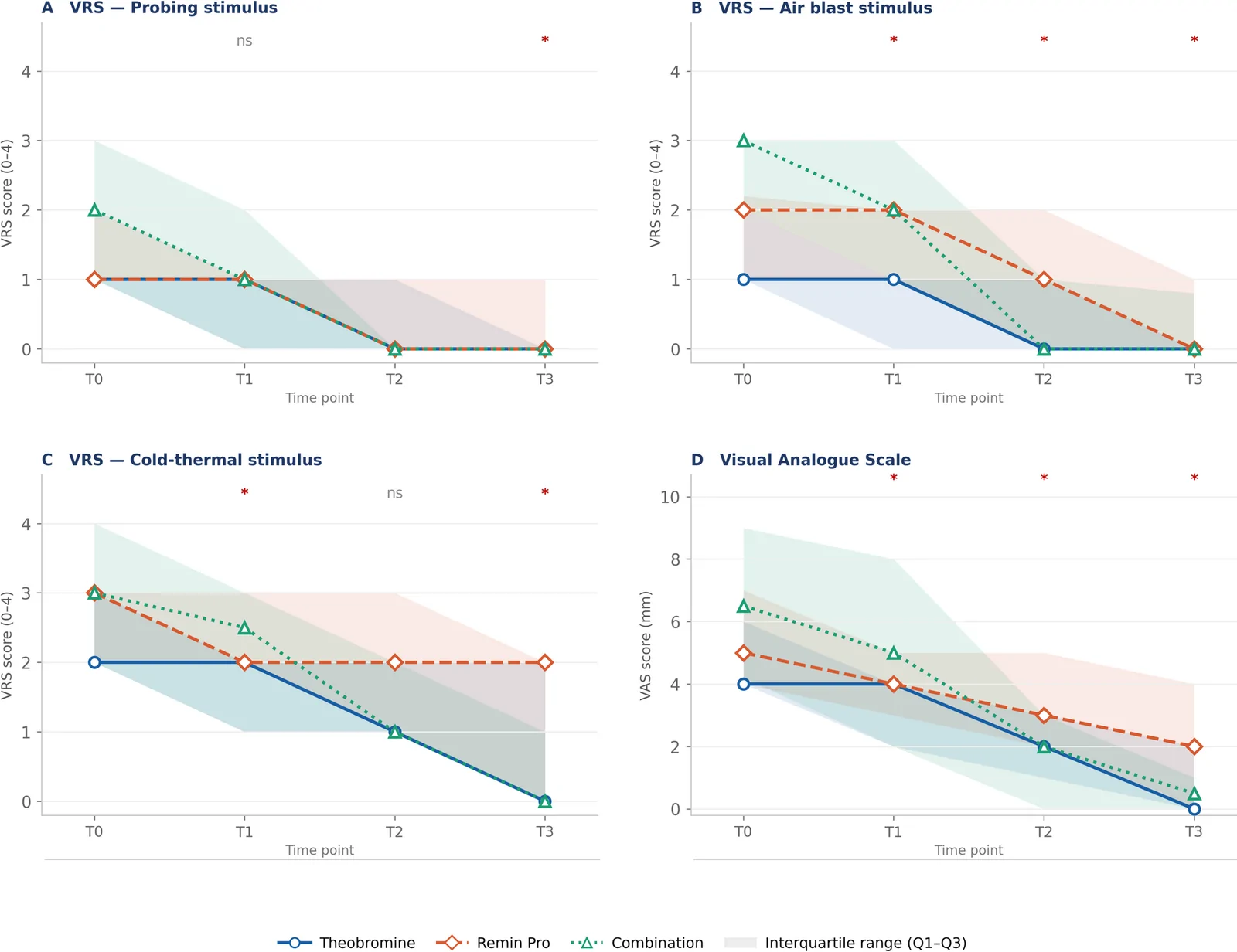
Median pain scores over time for the three treatment groups across four outcome measures. T0 = baseline (before treatment); T1 = immediately after first application; T2 = after second application (1 week); T3 = after third application (1 month). Lines represent median scores; shaded bands represent the interquartile range (Q1–Q3). VRS = Verbal Rating Scale (0–4); VAS = Visual Analogue Scale (0–100 mm). * *p* < 0.05 (Kruskal-Wallis test with Dunn’s post-hoc correction); ns = non-significant

Statistically significant intragroup reductions in pain scores were observed across all groups and outcomes (*p* < 0.001), confirming that all three treatment modalities produced progressive and meaningful reductions in dentin hypersensitivity over the study period. Results are expressed as median [IQR]. The temporal trajectory of pain score reduction across all four outcome measures is illustrated in Fig. 1.

#### VRS probing stimulus

At T1 (immediate), intergroup differences in VRS-probing scores were non-significant (H = 5.08, *p* = 0.079), indicating equivalent immediate effectiveness for all three agents in response to tactile stimulation. Significant differences emerged at T2 (H = 15.06, *p* < 0.001), where the combination group achieved significantly lower scores than Remin Pro (Bonferroni-corrected *p* = 0.001). At T3, Remin Pro showed higher residual scores than the combination group (*p* = 0.007), while theobromine was intermediate. Intragroup Friedman’s test confirmed a significant progressive reduction over time in all groups (*p* < 0.001). Full data are presented in Table 2.

**Table 2:** VRS Probing Stimulus: Median [IQR] and intergroup comparisons

Time Point	Theobromine Median [IQR]	Remin Pro Median [IQR]	Combination Median [IQR]	K-W *p*-value
T0 (Baseline)	1.0 [1.0–1.0]	1.0 [1.0–2.0]	2.0 [1.0–3.0]	< 0.001*
T1 (Immediate)	1.0 [0.0–1.0]	1.0 [1.0–1.0]	1.0 [0.0–2.0]	0.079 ns
T2 (1 Week)	0.0 [0.0–1.0]	0.0 [0.0–1.0]	0.0 [0.0–0.0]	< 0.001*
T3 (1 Month)	0.0 [0.0–0.0]	0.0 [0.0–1.0]	0.0 [0.0–0.0]	0.007*
Friedman p	< 0.001*	< 0.001*	< 0.001*	—

*IQR *= interquartile range, *K-W* = Kruskal-Wallis, * p < 0.05, *ns* = non-significant. Post-hoc Dunn's (Bonferroni): T2 — Remin Pro vs Combination: p = 0.001*; T3 — Remin Pro vs Combination: p = 0.007*

#### VRS air blast stimulus

The evaporative stimulus produced the most discriminating intergroup findings. At T1, theobromine showed significantly lower pain scores than both Remin Pro (*p* < 0.001) and the combination group (*p* < 0.001). This advantage persisted at T2, where theobromine remained superior to Remin Pro (*p* < 0.001), while the combination group was equivalent to theobromine and superior to Remin Pro alone. At T3, a residual significant difference between theobromine and Remin Pro was maintained (*p* = 0.007). Full data are presented in Table 3.

**Table 3:** VRS air blast stimulus: median [IQR] and intergroup comparisons

Time Point	Theobromine Median [IQR]	Remin Pro Median [IQR]	Combination Median [IQR]	K-W *p*-value
T0 (Baseline)	1.0 [1.0–2.0]	2.0 [1.0–2.2]	3.0 [2.0–3.0]	< 0.001*
T1 (Immediate)	1.0 [0.0–1.0]	2.0 [1.0–2.0]	2.0 [1.0–3.0]	< 0.001*
T2 (1 Week)	0.0 [0.0–1.0]	1.0 [0.0–2.0]	0.0 [0.0–1.0]	< 0.001*
T3 (1 Month)	0.0 [0.0–0.0]	0.0 [0.0–1.0]	0.0 [0.0–0.8]	0.005*
Friedman p	< 0.001*	< 0.001*	0.010*	—

Post-hoc Dunn's (Bonferroni): T1 — Theobromine vs Remin Pro: p < 0.001*, Theobromine vs Combination: p < 0.001*, Remin Pro vs Combination: ns. T2 — Theobromine vs Remin Pro: p < 0.001*, Remin Pro vs Combination: p < 0.001*, Theobromine vs Combination: ns. T3 — Theobromine vs Remin Pro: p = 0.007*

#### VRS cold-thermal stimulus

Cold-thermal stimulation produced the most persistent pain scores across all groups. At T1, significant intergroup differences were observed (H = 11.56, *p* = 0.003): theobromine showed lower scores than both Remin Pro (*p* = 0.011) and the combination (*p* = 0.007). The difference resolved by T2 (*p* = 0.050, ns), but re-emerged at T3, where Remin Pro showed significantly higher residual cold sensitivity than both theobromine (*p* = 0.001) and the combination (*p* = 0.001). Results are in Table 4.

**Table 4:** VRS Cold-Thermal Stimulus: Median [IQR] and intergroup comparisons

Time Point	Theobromine Median [IQR]	Remin Pro Median [IQR]	Combination Median [IQR]	K-W *p*-value
T0 (Baseline)	2.0 [2.0–3.0]	3.0 [2.0–3.0]	3.0 [2.0–4.0]	< 0.001*
T1 (Immediate)	2.0 [1.0–2.0]	2.0 [2.0–3.0]	2.5 [1.0–3.0]	0.003*
T2 (1 Week)	1.0 [1.0–2.0]	2.0 [1.0–3.0]	1.0 [1.0–2.0]	0.050 ns
T3 (1 Month)	0.0 [0.0–2.0]	2.0 [0.0–2.0]	0.0 [0.0–1.0]	< 0.001*
Friedman p	< 0.001*	< 0.001*	0.008*	—

Post-hoc Dunn's (Bonferroni): T1 — Theobromine vs Remin Pro: p = 0.011*; Theobromine vs Combination: p = 0.007*; Remin Pro vs Combination: ns. T3 — Remin Pro vs Theobromine: p = 0.001*; Remin Pro vs Combination: p = 0.001*

### Visual analogue scale

Visual Analogue Scale scores mirrored the Verbal Rating Scale findings. At T1, significant intergroup differences emerged (H = 7.98, *p* = 0.019), with theobromine achieving lower scores than the combination group (Bonferroni-corrected *p* = 0.016); the difference between theobromine and Remin Pro did not reach significance after Bonferroni correction (*p* = 0.182 ns). At T2, Remin Pro showed significantly higher residual scores than the combination group (*p* = 0.002). At T3, Remin Pro retained the highest median score (2.0 [0.0–4.0]) compared with theobromine (0.0 [0.0–2.0]; *p* = 0.003) and the combination (0.5 [0.0–1.0]; *p* < 0.001). Intragroup reductions were highly significant in all groups (*p* < 0.001). Full data are presented in Table 5.

**Table 5:** Visual Analogue Scale: Median [IQR] and intergroup comparisons

Time Point	Theobromine Median [IQR]	Remin Pro Median [IQR]	Combination Median [IQR]	K-W *p*-value
T0 (Baseline)	4.0 [4.0–6.0]	5.0 [4.0–7.0]	6.5 [4.2–9.0]	< 0.001*
T1 (Immediate)	4.0 [2.0–4.0]	4.0 [3.0–5.0]	5.0 [2.0–8.0]	0.019*
T2 (1 Week)	2.0 [1.0–3.0]	3.0 [2.0–5.0]	2.0 [0.0–3.0]	0.002*
T3 (1 Month)	0.0 [0.0–2.0]	2.0 [0.0–4.0]	0.5 [0.0–1.0]	< 0.001*
Friedman p	< 0.001*	< 0.001*	< 0.001*	—

Post-hoc Dunn's (Bonferroni): T1 — Theobromine vs Combination: p = 0.016*, others ns. T2 — Remin Pro vs Combination: p = 0.002*, others ns. T3 — Theobromine vs Remin Pro: p = 0.003*, Remin Pro vs Combination: p < 0.001*, Theobromine vs Combination: ns

## Discussion

The null hypothesis of this study, that no significant difference in dentin hypersensitivity reduction would be observed among the three tested agents at any assessment time point, was partially rejected. Statistically significant intergroup differences were detected at T1 and T2 for Verbal Rating Scale-air blast and Verbal Rating Scale-cold stimuli, and at T2 and T3 for Visual Analogue Scale scores, consistently demonstrating that theobromine showed early superiority over Remin Pro. For the probing stimulus, the null hypothesis was accepted at T1 (*p* = 0.079 ns), and the remaining difference at T3 (*p* = 0.007) was statistically significant but carried negligible clinical magnitude, with both theobromine and combination groups reaching a median of 0.0. The overall pattern of findings, most clearly illustrated in Fig. 1, supports the clinical interpretation that theobromine accelerates the early phase of tubule occlusion, while all three agents achieve comparable effectiveness after three applications for most outcomes.

The use of three mechanistically distinct stimuli, tactile, evaporative (air blast), and cold-thermal, was deliberate and methodologically sound. Each activates dentinal fluid flow via distinct biophysical mechanisms and recruits distinct sensory fiber populations [3, 23]. The evaporative stimulus was the most discriminating, producing the widest intergroup separation, consistent with evidence that air-blast stimulation is the most reliable probe of tubule patency in clinical desensitization research [19, 20]. Stimulus application followed an ascending-intensity sequence with a minimum 2-minute inter-stimulus interval, in accordance with current consensus recommendations to minimize sensory summation and carryover effects [18]. The use of both the Verbal Rating Scale and the Visual Analogue Scale as complementary instruments strengthened the convergent validity of pain measurement. Critically, both scales yield ordinal data that must be analyzed nonparametrically, a requirement that was not met in the original submission and is fully corrected in this revision.

The early desensitizing advantage of theobromine is consistent with in vitro evidence. Amaechi et al. [10] demonstrated that theobromine-containing toothpaste produced rapid and extensive dentinal tubule occlusion, with mineral plugs showing superior resistance to acidic challenge compared with fluoride-only controls. The mechanism involves promotion of calcium-phosphate crystallite nucleation to form larger, mechanically stable mineral deposits within the tubule lumen [9]. Kargul et al. [24] further reported a measurable increase in surface microhardness following theobromine application, suggesting a broader mineralization-promoting effect.

Remin Pro also produced significant and progressive pain reduction across all three stimulus categories, consistent with earlier randomized clinical trials on hydroxyapatite-based formulations [7, 25–27]. The mechanism of calcium fluoride deposition, as described by Petersson [6], operates through a fluoride-calcium ion exchange at the dentin surface, producing a sustained mineral reservoir that progressively reduces tubule hydraulic conductance. The slower onset of Remin Pro efficacy compared with theobromine is most evident at T1 for the air blast stimulus (Fig. 1B; median VRS: 2.0 [1.0–2.0] vs. 1.0 [0.0–1.0]) and likely reflects differences in crystallite nucleation kinetics and the time required for hydroxyapatite crystal integration into the tubule smear layer [28].

The persistent elevation of Remin Pro scores at T3 on the Visual Analogue Scale (Fig. 1D; median 2.0 [0.0–4.0] vs. 0.0 [0.0–2.0] for theobromine) and the wider interquartile range reflect greater inter-tooth variability in therapeutic response, possibly related to individual differences in tubule diameter, dentinal fluid flow rate, or surface smear layer composition [29]. This heterogeneity was not apparent in the theobromine group, which showed consistently low median scores at T3 across all stimuli.

The combination of theobromine and Remin Pro produced outcomes generally intermediate between the two individual agents, without consistently exceeding either. At T2 for the air blast stimulus, the combination was equivalent to theobromine and superior to Remin Pro alone, suggesting at least an additive contribution of theobromine’s early crystallite-forming action. The absence of a clear synergistic advantage over theobromine alone may reflect physicochemical interactions when the two materials are mixed, including potential dilution of theobromine concentration or competitive crystal nucleation at the tubule orifice [30].

This study has several notable strengths: prospective pre-registration (ClinicalTrials.gov; NCT04340869), double-blind design with allocation concealment, standardized multi-stimulus assessment protocol, dual complementary pain scales, calibrated outcome assessor (κ = 0.85), pH-monitored theobromine gel preparation, and a complete re-analysis of raw ordinal data using appropriate nonparametric statistics with Bonferroni-corrected post-hoc testing. Furthermore, the baseline assessment (T0), recorded before any treatment was administered, serves as a within-subject internal control for each individual tooth, making this a pre-post-controlled design. The Friedman test is specifically structured for this repeated-measures framework, treating each tooth as its own control across time.

The following limitations must be acknowledged. First, the one-month follow-up period is short, and dentin hypersensitivity may relapse after dissolution of the mineral plug by salivary or dietary acids; longer-term studies (≥ 6 months) are needed to determine durability. Second, all participants were female, limiting generalizability. Third, clustering of multiple teeth within 20 patients creates intraclass correlation that is not formally modeled in the analysis; future studies should use mixed-effects models or generalized estimating equations with patient as a random effect. Fourth, the absence of an external placebo arm, justified on ethical grounds because withholding active treatment from symptomatic patients was not approved by the institutional ethics committee, prevents full isolation of the treatment-specific effect from natural remission or placebo response; future trials incorporating a neutral carrier gel arm are recommended. Fifth, dietary acid compliance could not be objectively verified.

## Conclusions

Within the inherent constraints of this short-term, single-center, randomized clinical study, the following conclusions are warranted:

1. Theobromine gel provides clinically and statistically significant faster desensitization than Remin Pro at early treatment time points, most consistently in response to evaporative and cold-thermal stimuli. This early advantage is directly relevant to patients presenting with acute or severe dentin hypersensitivity who require prompt symptom control.
2. After three sequential applications over one month, theobromine gel, Remin Pro, and their combination achieve broadly comparable clinical effectiveness in reducing dentin hypersensitivity across multiple stimuli, confirming that a multi-application protocol is the standard of care regardless of agent selection.
3. Remin Pro demonstrated greater inter-patient variability in therapeutic response, particularly at the final assessment, which may be relevant for clinical agent selection and patient stratification.

These findings support the use of theobromine gel as a viable, biocompatible first-line desensitizing agent, particularly when rapid symptom relief is the primary objective, and justify further investigation in long-term, placebo-controlled, multi-center randomized trials with larger and more diverse patient populations.

## Supplementary Information

Supplementary Material 1.

Supplementary Material 2.

Supplementary Material 3.

Supplementary Material 4.

Supplementary Material 5.

## Data Availability

The data presented in this study are available upon request from the authors.
