# Supplementary material for: Clinical effectiveness of theobromine, Remin Pro, and their combination in managing dentin hypersensitivity: a randomized clinical trial
Source: BMC Oral Health. 2026 Oct 2;26:1923. doi: 10.1186/s12903-026-09869-0 (PMC13637199; doi:10.1186/s12903-026-09869-0)
Supplement: Supplementary file 1 [file 12903_2026_9869_MOESM1_ESM.pdf]

RESEARCH ETHICS COMMITTEE
FACULTY OF DENTAL MEDICINE for Girls
AL-AZHAR UNIVERSITY

Principal Investigators: Ebtsam E. Kassab, Maha A. Niazy, Mohammed A. El-Yasky, Shimaa Ameen

Department: Postdoctoral Researchers

**Ethics Committee Board Decision**
The Research Ethics Committee of the Faculty of Dental Medicine for Girls, Al-Azhar University is constituted and operates according to ICH GCP guidelines and applicable local and Institutional regulations and guidelines which govern IRB operation. The committee met in February 2026.
**Study Title: Clinical effectiveness of theobromine, Remin Pro®, and their combination in managing dentin hypersensitivity: a clinical study**
**Protocol Code**: **P-OP-18-02**
**Final Code**: **REC-OP-26-01**

Dear Dr.

The Research Ethics Committee, Faculty of Dental Medicine for Girls, Al-Azhar University met and reviewed the presented documents after completing the research which in consensus with the data obtained from the basic study protocol and, this is a final research approval for documentation and publication proposes.

Yours sincerely,

Chairman of Research Ethics Committee

Mervat fawzy

رقم التسجيل:
Registration Number:
SCCREIRB-DNTLMEDICINGRLSAZHRCAIRO-GU-001-270525-068

Address: Faculty of Dental Medicine for Girls, Al-Azhar University, Nasr City.
Email: ethics.committee@azhar.edu.eg /
azhardentistry.rec@gmail.com
