# Supplementary material for: Clinical effectiveness of theobromine, Remin Pro, and their combination in managing dentin hypersensitivity: a randomized clinical trial
Source: BMC Oral Health. 2026 Oct 2;26:1923. doi: 10.1186/s12903-026-09869-0 (PMC13637199; doi:10.1186/s12903-026-09869-0)
Supplement: Supplementary file 2 [file 12903_2026_9869_MOESM3_ESM.pdf]

جامعه الازهر
كليه طب الاسنان
فرع البنات

<u>الموافقه المستنيره للمتطوعين</u>

عنوان البحث باللغة العربية:

تأثير استخدام مثبطين احساس كل على حدة وخليط منهما على نفاذية وفرط حساسية عاج الأسنان.

الهدف من إجراء البحث:

هذه الدراسة لتقييم ومقارنة التأثير الاكلينيكى لمثبطات حساسية عاج الأسنان المختلفة.

خطة العمل:

سيتم اختيار المرضى الذين يعانون من فرط حساسية الاسنان وصحتهم العامة جيدة وتتراوح أعمارهم بين 18-35 سنة.

سيتم تقسيم المرضى عشوائيا الى ثلاث مجموعات يتم فيها معالجة المرضى الذين يعانون من فرط حساسيه عاج الأسنان إما باستخدام الثيوبرومين أو ريمين برو أو باستخدام كلاهما معا.

سيتم معالجة العاج المصاب موضعيا وسيتم تسجيل مستوى فرط حساسية عاج الأسنان باستخدام مقياس التماثل البصرى قبل البدء فى المعالجة وبعد المعالجة فورا ثم بعد ٧ أيام ثم بعد ثلاثون يوما من المعالجة.

سيتم تجميع النتائج وجدولتها وتحليلها إحصائيا.

الفائدة المباشرة للشخص المتطوع:

أنه سوف يتلقى علاجا لمشكلة فرط حساسية عاج الاسنان.

الفائدة العلمية والفائدة العامة المرجوة من البحث:

تقييم ومقارنة التأثير الاكلينيكى لمثبطات حساسية عاج الأسنان المختلفة بطريقة علمية منظمة وممنهجة.

الاعراض الجانبية ودرجة المخاطر المتوقع حدوثها وكيفية التعامل معها:

سوف يتم استبعاد المرضى الذين يعانون من حساسية لمثبطات حساسية عاج الاسنان المستخدمة فى الدراسة او لأى من مكوناتها

<u>المعرفه الكامله للمريض بخطوات البحث:</u>

قراءة [ ]   شرح شفهي [ ]   أخرى [ ].

١. لقد اطلعت بعناية وفهمت الغرض من إجراء البحث وطبيعة هذه الدراسة، وأنا أفهم ما هو ضروري لإنجاز هذه الإجراءات.

٢. قد أعلمنى الطبيب الباحث بالبدائل العلاجية الممكنة لهذا البحث.

٣. لقد أبلغنى الطبيب الباحث بجميع المخاطر المحتملة لهذا البحث وكيفية التعامل معها.

٤. أوافق على التصوير والتسجيل والذي يتعين القيام به فى هذا الدراسة، بشرط عدم الكشف عن هويتي.

٥. لقد قدمت تقريرا دقيقا عن تاريخ حالتى الصحية. وأبلغت الطبيب بجميع أنواع ردود الأفعال الصحية أو الحساسية غير العادية من الأدوية أوالأغذية أولدغ الحشرات أومواد التخدير أوالغبار أو أى ردود

أفعال حدثت لى من أى مواد أخرى، أو نزيف غير طبيعي أو أي ظروف أخرى ذات صلة على صحتي

٦. أقر بأنني غير مشترك في أى بحث أخر منذ بداية هذا البحث وحتى إنتهائه وأننى سأعلم الطبيب الباحث لو دخلت أى بحث أخر طوال فترة هذا البحث.

بعد معرفة المعلومات المتاحة الخاصة بالبحث يتفضل الشخص المتطوع أو المسئول عنه بالاختيار بحرية ما بين الاشتراك من عدمه. في حال الموافقة يتفضل بملء البيانات الموضحة . من حق المتطوع الإنسحاب من البحث بدون إبداء الأسباب.

تعهد الطبيب المسئول عن البحث بالحفاظ على سرية المعلومات الخاصة بالشخص المتطوع بالمشاركة في البحث مع ذكر الطرق المستخدمه لذلك مثل استبدال الاسماء بارقام كوديه أو اخفاء معالم الوجه عند التصوير الفوتوغرافي ان امكن (الخ..)

طريقة الاتصال في حالة أي شكوى

ت: ٠١٠٠١٧٤٨١٩١

ايميل: eshoreibah@yahoo.com

من حق المتطوع الاحتفاظ بنسخه مصورة من الموافقه المستنيرة للبحث الذي تطوع فيه

اسم المتطوع: تاريخ الميلاد:

الرقم القومي (ان وجد):

اسم ولي الأمر أو المرافق (عند اللزوم): الرقم القومي:

العنوان: الهاتف:

التاريخ:

اسم الباحث

توقيع الباحث: التاريخ

توقيع المشرف على البحث (في حالة الرسائل):

التاريخ:

بيانات تملأ بمعرفة اللجنة

هذا البحث تمت موافقة اللجنه عليه برقم

Approval code: REC18-145
