# Supplementary material for: Clinical effectiveness of theobromine, Remin Pro, and their combination in managing dentin hypersensitivity: a randomized clinical trial
Source: BMC Oral Health. 2026 Oct 2;26:1923. doi: 10.1186/s12903-026-09869-0 (PMC13637199; doi:10.1186/s12903-026-09869-0)
Supplement: Supplementary file 3 [file 12903_2026_9869_MOESM4_ESM.pdf]

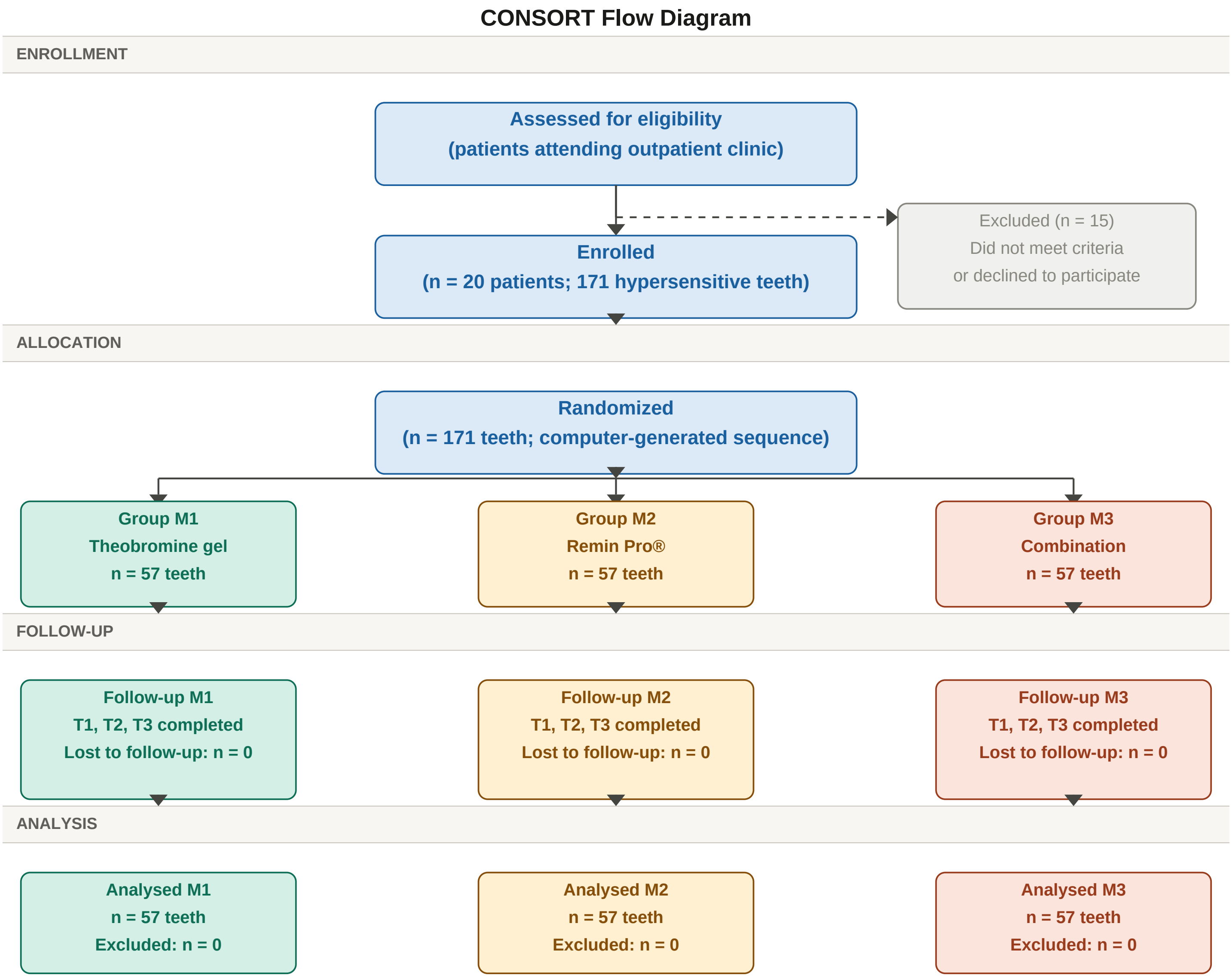

T0 = baseline; T1 = immediately after first application; T2 = 1 week after second application; T3 = 1 month after third application.
Dashed arrow = patients excluded after screening. Exclusion reasons: failure to meet one or more inclusion criteria, or refusal to participate.
Randomization unit: tooth level, using a computer-generated sequence. Allocation concealment via sealed opaque envelopes.
