# Supplementary material for: Clinical effectiveness of theobromine, Remin Pro, and their combination in managing dentin hypersensitivity: a randomized clinical trial
Source: BMC Oral Health. 2026 Oct 2;26:1923. doi: 10.1186/s12903-026-09869-0 (PMC13637199; doi:10.1186/s12903-026-09869-0)
Supplement: Supplementary file 4 [file 12903_2026_9869_MOESM5_ESM.docx]

**CONSORT 2010 Checklist**

*Clinical effectiveness of theobromine, Remin Pro®, and their combination in managing dentin hypersensitivity: a clinical study*

Kassab EE, Niazy MA, El Yasky MA, Ameen SM

ClinicalTrials.gov: NCT04340869

Journal: BioMed Central (BMC Series)

| **Section / Topic** | **Item No.** | **Checklist Item** | **Reported?** | **Page No.** |
| --- | --- | --- | --- | --- |
| **TITLE & ABSTRACT** | | | | |
| Title | 1a | Identification as a randomized trial in the title | Yes | 1 |
| Abstract | 1b | Structured summary of trial design, methods, results, and conclusions (for specific guidance, see CONSORT for abstracts) | Yes | 1 |
| **INTRODUCTION** | | | | |
| Background & objectives | 2a | Scientific background and explanation of rationale | Yes | 2 |
|  | 2b | Specific objectives or hypotheses | Yes | 2 |
| **METHODS** | | | | |
| Trial design | 3a | Description of trial design (such as parallel, factorial), including allocation ratio | Yes | 3 |
|  | 3b | Important changes to methods after trial commencement (such as eligibility criteria), with reasons | N/A | N/A |
| Participants | 4a | Eligibility criteria for participants | Yes | 3–4 |
|  | 4b | Settings and locations where the data were collected | Yes | 3 |
| Interventions | 5 | The interventions for each group should include sufficient details to allow replication, including how and when they were actually administered | Yes | 4–5 |
| Outcomes | 6a | Completely defined pre-specified primary and secondary outcome measures, including how and when they were assessed | Yes | 5 |
|  | 6b | Any changes to trial outcomes after the trial commenced, with reasons | N/A | N/A |
| Sample size | 7a | How the sample size was determined | Yes | 3 |
|  | 7b | When applicable, an explanation of any interim analyses and stopping guidelines | N/A | N/A |
| **RANDOMIZATION** | | | | |
| Sequence generation | 8a | Method used to generate the random allocation sequence | Yes | 4 |
|  | 8b | Type of randomization; details of any restriction (such as blocking and block size) | Yes | 4 |
| Allocation concealment mechanism | 9 | Mechanism used to implement the random allocation sequence (such as sequentially numbered containers), describing any steps taken to conceal the sequence until interventions were assigned | Yes | 4 |
| Implementation | 10 | Who generated the random allocation sequence, who enrolled participants, and who assigned participants to interventions? | Yes | 4 |
| Blinding | 11a | If done, who was blinded after assignment to interventions (for example, participants, care providers, those assessing outcomes) and how | Yes | 4 |
|  | 11b | If relevant, description of the similarity of interventions | N/A | N/A |
| Statistical methods | 12a | Statistical methods used to compare groups for primary and secondary outcomes | Yes | 5 |
|  | 12b | Methods for additional analyses, such as subgroup analyses and adjusted analyses | N/A | N/A |
| **RESULTS** | | | | |
| Participant flow (diagram strongly recommended) | 13a | For each group, the number of participants who were randomly assigned, received intended treatment, and were analyzed for the primary outcome | Yes | 6 + Flow Diagram |
|  | 13b | For each group, losses and exclusions after randomization, together with reasons | Yes | 6 + Flow Diagram |
| Recruitment | 14a | Dates defining the periods of recruitment and follow-up | Yes | 3 |
|  | 14b | Why the trial ended or was stopped | N/A | N/A |
| Baseline data | 15 | A table showing baseline demographic and clinical characteristics for each group | Yes | 6 |
| Numbers analyzed | 16 | For each group, the number of participants (denominator) included in each analysis, and whether the analysis was by the original assigned groups | Yes | 6 |
| Outcomes and estimation | 17a | For each primary and secondary outcome, results for each group, and the estimated effect size and its precision (such as 95% confidence interval) | Yes | 6–9 |
|  | 17b | For binary outcomes, presentation of both absolute and relative effect sizes is recommended | N/A | N/A |
| Ancillary analyses | 18 | Results of any other analyses performed, including subgroup analyses and adjusted analyses, distinguishing pre-specified from exploratory | N/A | N/A |
| Harms | 19 | All-important harms or unintended effects in each group | N/A | N/A |
| **DISCUSSION** | | | | |
| Limitations | 20 | Trial limitations, addressing sources of potential bias, imprecision, and, if relevant, multiplicity of analyses | Yes | 10 |
| Generalizability | 21 | Generalizability (external validity, applicability) of the trial findings | Yes | 10 |
| Interpretation | 22 | Interpretation consistent with results, balancing benefits and harms, and considering other relevant evidence | Yes | 9–10 |
| **OTHER INFORMATION** | | | | |
| Registration | 23 | Registration number and name of trial registry | Yes | 1 & 3 |
| Protocol | 24 | Where the full trial protocol can be accessed, if available | N/A | N/A |
| Funding | 25 | Sources of funding and other support (such as supply of drugs); role of funders | Yes | 10 |

** N/A = Not applicable to this trial design.*

*Citation: Hopewell S, Chan AW, Collins GS, Hróbjartsson A, Moher D, Schulz KF, et al. CONSORT 2025 Statement: updated guideline for reporting randomised trials. BMJ. 2025; 388: e081123.* [*https://dx.doi.org/10.1136/bmj-2024-081123*](https://dx.doi.org/10.1136/bmj-2024-081123)*.*
